# Determinants of COVID-19 vaccination intention in Central and Eastern Europe: A cross-sectional study in Poland, Romania, and Slovenia

**DOI:** 10.1186/s13690-024-01261-0

**Published:** 2024-04-30

**Authors:** Ana Slavec, Magdalena Iwanowska, Katarzyna Bałandynowicz-Panfil, Șerban Olah, Mojca Šoštarič Zvonar, Janez Štebe, Małgorzata Łosiewicz

**Affiliations:** 1 InnoRenew CoE, Izola, Slovenia and Faculty of Mathematics, Natural Sciences and Information Technologies, University of Primorska, Koper, Slovenia; 2 Institute of Psychology, Faculty of Social Sciences, University of Gdańsk, Gdańsk, Poland; 3 Department of Sustainable Market Processes, Faculty of Economics, University of Gdańsk, Gdańsk, Poland; 4 Department of Sociology and Social Work, University of Oradea, Oradea, Romania; 5 University Psychiatric Clinic Ljubljana, Ljubljana, Slovenia; 6 Social Science Data Archives (ADP), Faculty of Social Sciences, University of Ljubljana, Ljubljana, Slovenia; 7 Institute of Media, Journalism and Social Communication, Faculty of Social Sciences, University of Gdańsk, Gdańsk, Poland

**Keywords:** COVID-19, Vaccination intention, Attitudes towards vaccines, Conspiracy mentality, Trust in information sources, Central and Eastern Europe

## Abstract

**Background:**

Identifying predictors of vaccination intention is critical to developing appropriate programs and campaigns targeting groups reluctant to be vaccinated. This study aimed to identify the determinants of vaccination intention at the beginning of the COVID-19 pandemic in three Central and Eastern European (CEE) countries: Poland, Romania, and Slovenia.

**Methods:**

In this cross-sectional study, a sample of unvaccinated 1723 Poles, Romanians, and Slovenians completed an online survey (April 2021). Questions included measures of vaccination intention, attitudes towards vaccines, conspiracy mindset, preference for a type of vaccine, and trust in information sources.

**Results:**

The results showed that mistrust of vaccine benefits and concerns about commercial profiteering negatively predicted vaccination intention. Conversely, trust in information from medical professionals and scientists, official sources, and traditional media was positively related to vaccination intention, while trust in digital media was negatively related to vaccination intention. In addition, preference for mRNA vaccine type was a positive significant predictor of vaccination intention. The differences between countries are discussed.

**Conclusions:**

The study results deliver suggestions for developing appropriate vaccine uptake programs and campaigns that should consider presenting the positive outcomes of vaccines via official sources and traditional media based on scientific evidence and medical professionals’ knowledge.

**Table:** 

**Text box 1. Contributions to the literature**
• Adding further empirical research data to better understand the relationship between attitudes towards vaccinations, conspiracy mentality, social trust, and vaccination intention.
• Communicating an observation on the importance of comparative cross-cultural studies in the realm of social behavior during epidemiological threat situations.
• Providing specific insights into the determinants of COVID-19 vaccination intention within the Central and Eastern European context, and offering practical implications for region-specific public health campaigns.

## Background

When the World Health Organization (WHO) announced the COVID-19 pandemic on March 11, 2020, governments and researchers began seeking solutions to prevent the spread of the disease caused by the new coronavirus, SARS-CoV-2. The first vaccine appeared in December 2020 [1]. However, the availability of vaccines did not guarantee widespread acceptance. Achieving herd immunity posed a significant challenge for healthcare systems worldwide, with a persistent high prevalence of hesitancy toward COVID-19, particularly in Central and Eastern Europe (CEE) [2–4]. The official starting date of inoculation for Romania was December 28, 2020, and for Poland and Slovenia, it was December 29, 2020. Four months after the introduction of vaccines, 16% of people had received at least one dose of the COVID-19 vaccine in Poland, 13% in Romania, and 17% in Slovenia (see Table 1).

**Table 1:** Polish, Romanian, and Slovenian COVID-19 vaccination rates in April and September 2021 [16]

	Poland	Romania	Slovenia
Population number	37 972 800	19 414 500	2 080 900
Confirmed COVID-19 cases per million people			
15.04.2021	69 906	53 341	110 629
15.09.2021	76 599	59 107	134 321
Share of people who had received at least one dose of COVID-19 vaccine			
15.04.2021	16.15%	12.92%	17.46%
15.09.2021	51.81%	27.91%	50.79%
Share of the population fully vaccinated against COVID-19			
15.04.2021	5.95%	7.98%	6.39%
15.09.2021	50.71%	27.34%	45.64%

Exploring factors associated with the acceptance of COVID-19 vaccination became an important research task. As indicated by researchers of vaccine hesitancy, when analysing the determinants of the intention to vaccinate, one should consider (1) individual influences that arise from personal attitudes and beliefs; (2) vaccine-specific influences directly related to the vaccine or vaccination; and (3) group influences from the social environment [3]. To the best of the authors’ knowledge, no cross-cultural studies on the determinants of COVID-19 vaccination intention have been conducted at the onset of the pandemic for countries in the Central and Eastern Europe region. The presented study addressed this gap by presenting a model of predictors that encompasses individual (i.e., attitudes toward vaccines and conspiracy mindset), vaccine-specific (i.e., preference for the vaccine type), and social (i.e., trust in information sources) factors influencing the intention to vaccinate against COVID-19 in Poland, Romania, and Slovenia. We examined predictors that could offer valuable insights to formulate recommendations for health policy decision-makers, aiming to develop more pertinent campaigns to promote vaccinations among groups reluctant to be vaccinated in CEE countries. Below, previous studies on the substantial predictors of vaccination intention in the COVID-19 context will be presented and formulated hypotheses will be introduced.

Individual factors, such as attitudes and beliefs, are dominant predictors of vaccination intention [4]. Although many studies confirm the positive effects of vaccines [5], the negative attitudes towards them may endanger the vaccination process in the long term. Attitudes towards vaccination are described as a continuum, ranging from complete acceptance to total rejection [3]. Attitudes towards vaccines include the following common factors: mistrust of vaccine benefits, worries about unforeseen future effects, concerns about commercial profiteering, and a preference for natural immunity [6]. In the COVID-19 pandemic, it has been shown that positive general attitudes towards vaccination can lead to intentions to vaccinate against COVID-19 [7], and therefore, we expected that: *negative attitudes towards vaccination predict negatively the intention to take the COVID-19 vaccine (Hypothesis H1a).*

Studies also indicate that adopting a conspiracy mentality, which is a predisposition to explain events as conspiracies [8], significantly impairs people’s intentions to get vaccinated [9]. Some authors even argue that antivaccination attitudes are part of a broader psychological tendency to believe in conspiracy theories [10]. Conspiracy theories can be defined as ‘attempts to explain the ultimate causes of significant social and political events and circumstances with claims of secret plots by two or more powerful actors’ [11]. These beliefs tend to rise in social crises when collective uncertainty and fear are high [12]. During the COVID-19 pandemic, various conspiracy theories regarding vaccines were spread, such as claims about their potential harm and the unfounded notion of modifying humans’ DNA [13]. Conspiracy mentality and conspiracy beliefs may be important predictors of unfavourable health behaviours, such as not accepting vaccination, as confirmed in the first COVID-19 studies [14, 15]. Thus, we assumed that: *conspiracy mentality negatively predicts the intention to take the COVID-19 vaccine (Hypothesis H1b).*

Another important factor influencing the decision to vaccinate is the preference for a specific vaccine type or manufacturer. During the current study, vaccines from four manufacturers were available in the European Union: Moderna mRNA-1273, Pfizer/BioNTech BNT162b2, Janssen (Johnson & Johnson) Ad26.COV2.S (one dose), and Oxford/AstraZeneca AZD1222,^1^ but there were also vaccines authorized outside the European Union or in advanced phases of clinical trials. Higher levels of trust were observed for the mRNA vaccine compared to the inactivated and live attenuated vaccines [17]. Therefore, we expected that: *the preference for mRNA vaccines positively predicts the intention to take the COVID-19 vaccine (Hypothesis H2).*

The influence of the social environment, as highlighted by various studies [4], also plays an important role in vaccination decisions. Depending on the information from the sources people trust, their intention to get vaccinated may vary. In an emergency, people search for information about a disease from multiple sources so that they can take appropriate action [18, 19]. The sources that have the greatest impact on medical decisions include relatives and people’s circles of friends. Their nearest and dearest or significant others are a source of trust in the vaccination system, its safety, and its effectiveness [20–22]. Studies have already demonstrated the impact of family and friends’ opinions on creating positive attitudes toward vaccination against COVID-19 [23]. However, in antivaccination circles, negative attitudes are likely reinforced by the opinions of families and friends as well. Some studies suggest that vaccine hesitancy is associated with greater trust in friends and family and reduced trust in doctors [24]. Therefore, we suspected that: *there is a relationship between trust in COVID-19 information from relatives and friends and the intention to take the COVID-19 vaccine (Hypothesis H3a).* As the existing findings are ambiguous, we did not hypothesize about the sign of the correlation coefficient.

Another group with whom individuals have direct, close, and fairly regular contact includes doctors, nurses, and other medical professionals. Physicians, with their level of knowledge, are identified as the main source of influence among the key determinants of vaccination [25–28]. Moreover, evidence-based knowledge and the promotion of confidence in science contribute to a decrease in anti-vaccination rates [29]. Therefore, we assumed that *trust in COVID-19 information from medical professionals and scientists positively predicts the intention to take the COVID-19 vaccine (Hypothesis H3b).*

Along with healthcare professionals, spiritual and religious leaders play a role in distributing immunization information– especially in traditional societies. While morally bound to lead their followers toward well-being by disseminating reliable information, they are also subject to a religious obligation. In Catholicism, the most morally questionable issue regarding vaccination is the use of cell lines derived from voluntarily aborted foetuses [30]. However, as most religions have no theological objection to vaccination, we assumed that *trust in COVID-19 information from religious leaders positively predicts the intention to take the COVID-19 vaccine (Hypothesis H3c).*

A high level of trust in official sources of information affects the level of perceived threat, the methods and sources of obtaining information, compliance with sanitary regimes, and making protective decisions. The lack of trust in the government and official sources is associated with undermining the credibility of information [31, 32], which may be particularly emphasized in sharply politically divided societies, as is the case in the region in this study [33]. That is why we assumed that *trust in COVID-19 information from official sources positively predicts the intention to take the COVID-19 vaccine (Hypothesis H3d).*

The media, which is an intermediary between public institutions and citizens, is also of great importance. Communication about public health takes place through various information channels. Some information channels are more credible, while others expose recipients to greater exposure to disinformation. First COVID-19 research indicates that people who trust information from traditional media are more likely to accept the vaccine than people who have more trust in social media [34]. Considering these research findings, we expected that *trust in COVID-19 information from traditional media positively predicts intention to take the COVID-19 vaccine (Hypothesis H3e), while trust in COVID-19 information from digital media negatively predicts intention to take the COVID-19 vaccine (Hypothesis H3f).*

Countries in the regions of Central and Eastern Europe exhibit distinctive social, economic, and political values and behaviors shaped by their historical and political opportunity structures and socio-economical contexts. Based on Hofstede and his cultural values framework [35], Poland, Slovenia, and Romania share the highest levels of uncertainty avoidance, but they differ in terms of individualism. Poland is more individualistic and Slovenia and Romania lean towards a more collectivistic orientation. Also, Romania has the highest level of power distance which measures the degree to which the members of a group or society accept the hierarchy of power and authority. Therefore, Romanian people accept a hierarchical order of society in which everybody has a place and which needs no further justification [35]. These differences may result in various attitudes towards vaccination [13, 36, 37]. We assume that, due to socio-economic and cultural discrepancies, different effects might be observed in these countries. For example, the positive relationship between trust in official sources and the intention to vaccinate should be higher in Romania.

## Method

### Purpose and design of the study

The study objectives were to estimate the rate of willingness to receive COVID-19 vaccination among the Polish, Romanian, and Slovenian populations, as well as to explore the determinants of the intention among unvaccinated individuals to receive a COVID-19 vaccine. Additionally, the study aimed to explore potential differences in these relationships in these three Central Eastern European countries. A cross-sectional study was conducted among the general population of unvaccinated individuals aged 18 and above in Poland, Romania, and Slovenia.

### Sample

In our research, we chose to focus exclusively on unvaccinated individuals to investigate the influencing factors within this majority at the time of the study. In total, 3952 people started responding the survey, but 2211 were already vaccinated so they were excluded from continuing. The remaining 1741 who were not vaccinated continued filling out the survey and of these 17 dropped out so the final of participants was 1723: 300 in Poland, 388 in Romania, and 1035 in Slovenia (before weighting: 52.2% of women, 47.8% of men, *M*_*age*_ = 45.29).

### Data collection

Data collection took place with an online survey conducted in April 2021. The survey in Poland and Slovenia was run through online marketing research panels based primarily on a probability sample from the national statistical office, while in Romania, the data were collected with snowball sampling through social media and other channels with the help of a group of students. The data used in this research are publicly available in the Slovenian Social Science Data Archive (ADP) [38].

The obtained data were post-stratification weighted to the population totals by gender and age. To make the results comparable, the multivariate analysis also included the total weight, which made the country sample sizes equal. The analysis of the data was done using IBM SPSS Statistics for Windows 21.0. We used regression analysis to study what factors affect if someone intends to get vaccinated or not. In Model 1 (M1) we only included trust in information sources (with sociodemographic factors), while in Model 2 (M2) we also included negative attitudes towards vaccination and conspiracy theory beliefs (individual factors) and preference for a type of vaccine (vaccine-specific factors) on the decision to vaccinate. Two enter logistic regression models were used instead of using a stepwise procedure which is sensitive to multicollinearity and might lead to overfitting.

All procedures performed in studies involving human participants were following the ethical standards of the institutional research committee and with the 1964 Helsinki Declaration and its later amendments or comparable ethical standards. Before commencing the study, we obtained informed consent from the participants. Participation was voluntary, and no incentives were offered to participants, except in Poland, where participants received panel points that could be converted into money.

### Measures

The questionnaire included scales measuring the intended variables and questions unrelated to this study.It was initially developed in English and subsequently translated into Polish, Romanian, and Slovenian. These translations were then back-translated into English to assess the quality of the translation. 

#### Vaccination intention

We asked participants how much they agreed with the statement that they would get vaccinated if the vaccine was available using a 7-point Likert scale from 1– *strongly disagree* to 7– *strongly agree*. As the observed variable based on this question is ordinal, we could not use it as the dependent variable in linear regression so we decided to run logistic regression, which required us to recode it to a dichotomous variable, where the categories *slightly agree* and the above were coded as 1 (*intends to vaccinate*), while the other categories were coded as 0 (*does not intend to vaccinate*).

#### Predictor variables

##### Attitudes on vaccination

We used a 12-item Vaccination Attitudes Examination (VAX) to measure attitudes toward vaccination [6]. Participants were asked to focus on vaccines in general, rather than specifically on the COVID-19 vaccine (e.g. *I feel safe after being vaccinated*). Responses were rated on a 7-point Likert scale from 1– *strongly agree* to 7– *strongly disagree*. Four subscales were calculated: (1) mistrust of vaccine benefits (α = 0.85), (2) worries about unforeseen future effects (α = 0.79), (3) concerns about commercial profiteering (α = 0.87), and (4) preference for natural immunity (α = 0.89). The higher the score (mean of all items), the more negative the attitudes toward vaccination.

##### Conspiracy mentality

To assess differences in the generic tendency to engage in conspiracist ideation, we used the Conspiracy Mentality Questionnaire [39]. It consists of 5 items (e.g. *I think…a number of important things happen in the world, which the public is never informed about*) rated on a 5-point Likert scale from 1– *definitely not true* to 5– *definitely true* that compose one scale (α = 0.81).

##### Preference for a type of vaccine

We measured the preference for a type of vaccine with the question, ‘Which vaccine product would you prefer to take if you could choose?’. The list of items included both vaccine manufacturers that were available in the country and those that were not. Each item was rated on a 5-point Likert scale from 1– *not prefer at all* to 5– *extremely prefer*. To be used as dependent variables in logistic regression, the observed variables based on this question were recoded. First, we computed the average value for the mRNA vaccines (Pfizer and Moderna) and the vector vaccines (AstraZeneca and Janssen), which were approved in EU countries. Second, we computed two composite variables: an exclusive preference for mRNA vaccines (an average above 1 for mRNA vaccines and an average of exactly 1 for vector vaccines) and a mixed preference (an average above 2.5 for both mRNA and vector vaccines).

##### Trust in COVID-19 information

To measure trust in COVID-19 information, we asked to what extent participants trusted the presented sources to get information about COVID-19. The list included different sources of information grouped into 6 categories: relatives and friends (α = 0.85), medical professionals and scientists (α = 0.78), official sources of information (α = 0.82), traditional media (α = 0.85), digital media e.g. social media (α = 0.65), and religious leaders– rated with one item. Each item was rated on a 6-point Likert scale from 1– *very little extent* to 5– *very great extent*; 6 was ‘don’t use that source of information at all’, which was recoded as 1– *very little extent*.

##### Socio-demographic variables

Socio-demographic variables were measured using multiple-choice items. Age was measured as a ratio scale and gender as a categorical scale. Location was assessed with a question about where the participants lived. Education level was assessed with a question about the last school from which the participant had graduated. Socioeconomic status was measured with MacArthur’s Scale of Subjective Social Status [40]. The political stance was measured with a question as to how the participant would describe their political stance on a 7-point Likert scale from 1– *far right* to 7– *far left*. Religious habits were measured with questions about the frequency of attending church or other religious meetings and time spent in private religious activities (α = 0.72). Participants answered the question on a scale from 1– *rarely or never* to 6– *more than once a day.* Intimate religious beliefs were computed as the average of the agreement of three items about experiencing the divine (α = 0.88). Participants answered using a 5-point Likert scale from 1– *definitely not true for me* to 5– *definitely true for me*.

## Results

**Fig. 1:**
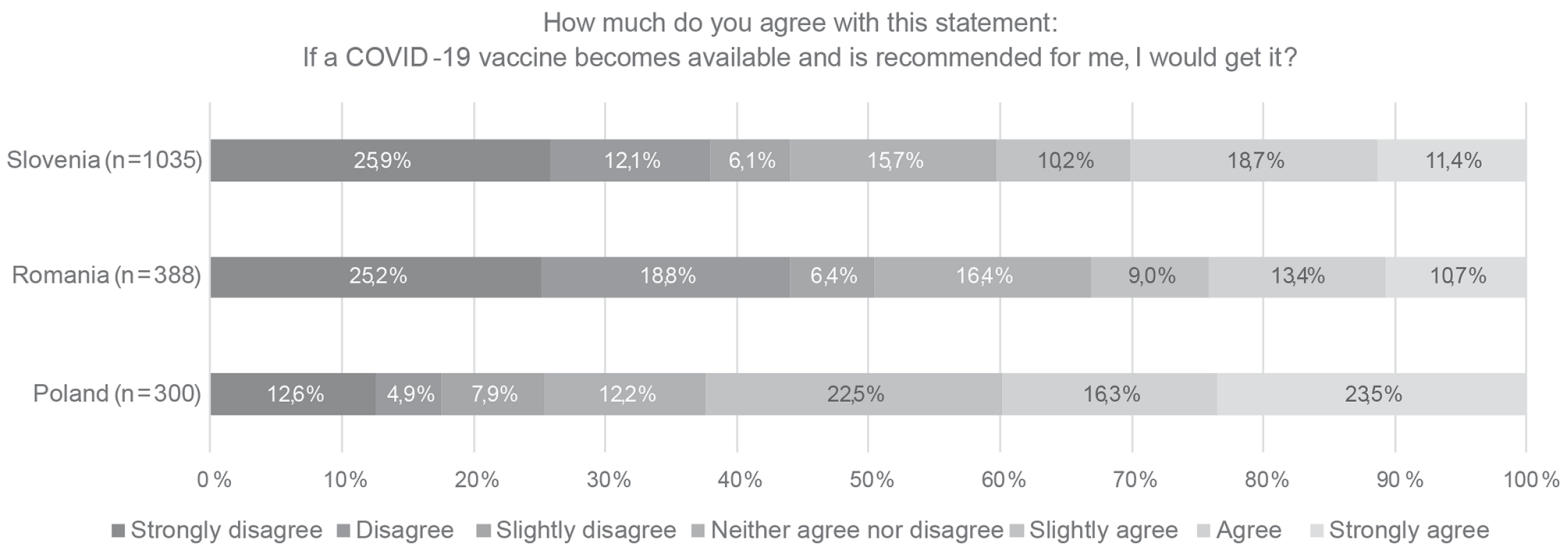
The response distributions for vaccination intention in the Polish, Romanian, and Slovenian samples

Figure 1 shows the response distributions for vaccination intention in the Polish, Romanian, and Slovenian samples.

According to our research 62.3% of Poles, 33.1% of Romanians, and 40.3% of Slovenians who were unvaccinated had the intention to get vaccinated, while 25.4% of Poles, 50.5% Romanians, and 44.1% Slovenians would not get vaccinated.

We present the descriptive statistics of the key variables in Table 2.

**Table 2:** The descriptive statistics of the key variables in the general sample and in each country - in this table mistrust... and worries.. are combined in one cell - they should be separate

	General (*n* = 1723)	Poland (*n* = 300)	Romania (*n* = 388)	Slovenia (*n* = 1035)
**Attitudes towards vaccination**				
Mistrust of vaccine benefit	4.45 (0.06)	3.6 (0.08)	4.86 (0.20)	4.53 (0.06)
Worries over unforeseen future effects	5.28 (0.03)	4.88 (0.07)	5.36 (0.07)	5.37 (0.04)
Concerns about commercial profiteering	4.61 (0.04)	3.86 (0.09)	4.73 (0.10)	4.79 (0.06)
Preference for natural immunity	4.48 (0.04)	4.16 (0.08)	4.85 (0.08)	4.42 (0.05)
**Conspiracy mentality**	3.74 (0.02)	3.82 (0.05)	3.59 (0.04)	3.78 (0.03)
**Trust in sources of information**				
Family and friends	3.10 (0.03)	3.28 (0.07)	2.83 (0.06)	3.15 (0.03)
Medical professionals & scientists	3.10 (0.03)	3.39 (0.06)	2.84 (0.05)	3.12 (0.03)
Religious leaders	1.73 (0.03)	1.98 (0.06)	1.98 (0.06)	1.56 (0.03)
Official sources	2.34 (0.03)	2.77 (0.06)	1.98 (0.05)	2.35 (0.03)
Traditional media	2.42 (0.03)	2.64 (0.06)	2.21 (0.05)	2.43 (0.03)
Digital media	2.19 (0.02)	2.55 (0.06)	1.95 (0.05)	2.17 (0.03)

Among the attitudes towards vaccination, the highest result was for worries about vaccines’ future effects in all countries. The mean for conspiracy mentality was moderate. Among the sources of information, family and friends, as well as medical and scientific sources, were the most trusted, while religious leaders were the least trusted. 

Table 3 presents the logistic regression results of the COVID-19 vaccination intention for the general sample and each country. We present two models. The first (M1) included only trust in information sources (with sociodemographic factors) as predictors of the decision to vaccinate., while the second (M2) also included negative attitudes towards vaccination and conspiracy mentality, and preference for a type of vaccine.

**Table 3:** Predictors of COVID-19 vaccination intention in the general sample and in each country

	General				Poland				Romania				Slovenia			
	M1		M2		M1		M2		M1		M2		M1		M2	
Variables	*B*		*B*		*B*		*B*		*B*		*B*		*B*		*B*	
Poland ^a^	0.95***		0.21													
Romania ^a^	0.29		0.20													
Gender ^b^	− 0.26		0.13		− 0.03		− 0.01		− 0.52		0.07		− 0.45		− 0.09	
Age	0.01		0.01*		0.00		0.00		0.03**		0.03*		0.01		0.02	
Higher education ^c^	0.28		0.13		0.23		− 0.01		0.31		− 0.12		0.21		0.18	
Living in a town or a city ^d^	0.09		− 0.31		− 0.21		− 0.64		0.32		− 0.90		0.06		0.02	
Socioeconomic status	0.00		− 0.07		0.07		− 0.03		− 0.16		− 0.24		− 0.01		− 0.07	
Political stance	− 0.10		− 0.01		− 0.06		− 0.03		− 0.12		− 0.18		0.02		0.16	
Religious habits	0.05		0.03		0.19		0.09		− 0.17		− 0.01		0.10		− 0.05	
Intimate religious beliefs	− 0.23**		− 0.08		− 0.28		− 0.09		− 0.26		− 0.05		− 0.05		0.04	
Trust in family and friends information	0.05		0.17		0.24		0.37		− 0.01		0.00		− 0.17		0.12	
Trust in medical professionals & scientists information	0.67***		0.11		0.47*		− 0.04		0.60**		0.07		0.96***		0.35	
Trust in religious leaders information	− 0.05		− 0.03		− 0.23		− 0.23		0.14		0.04		0.00		0.17	
Trust in official sources information	0.34**		− 0.06		0.12		− 0.25		0.67***		0.37		0.52*		0.02	
Trust in traditional media information	0.42***		0.26*		0.52**		0.44		0.51**		0.50		0.25		− 0.17	
Trust in digital media information	− 0.19*		− 0.09		− 0.15		− 0.05		− 0.03		− 0.01		− 0.41*		− 0.01	
Conspiracy mentality			0.17				0.50				− 0.27				0.05	
Mistrust of vaccine benefits			− 0.89***				− 0.93***				-1.09***				-1.01***	
Worries over unforeseen future effects			− 0.04				0.03				0.00				− 0.24	
Concerns about commercial profiteering			− 0.34**				− 0.69**				− 0.05				− 0.14	
Preference for natural immunity			− 0.05				0.14				0.16				− 0.33	
mRNA vaccine type preference			1.07**				4.06*				0.62				0.78	
Mixed preference for the vaccine type			1.68***				4.61**				2.27***				0.82	
Constant	-3.76***		2.16*		-2.25***		− 0.49		-4.19***		2.33		-4.29***		3.78	
Pseudo R^2^	*c*	*n*	*c*	*n*	*c*	*n*	*c*	*n*	*c*	*n*	*c*	*n*	*c*	*n*	*c*	*n*
	0.27	0.36	0.47	0.63	0.19	0.26	0.42	0.57	0.32	0.44	0.50	0.68	0.28	0.37	0.50	0.68

Note. Three countries together. Weighted as an equal sample size *N* = 300 each.

Significance level: * *p* < 0.05. ** *p* < 0.01. *** *p* < 0.001

M1– Model 1, M2– Model 2

c = Cox & Snell R^2^, n = Nagelkerke R^2^

^a^ Slovenia as reference. ^b^ Male as reference. ^c d^ Other as reference

The results of the regression in M1 indicated satisfactory goodness of fit (Cox-Snell *R*^*2*^ = 0.27; Nagelkerke *R*^*2*^ = 0.36, Model χ^2^ = 276.378, df = 16, *p* < 0.001). It was found that trust in information from medical professionals and scientists was the strongest predictor of vaccination intention (*B* = 0.67, *p* < 0.001). This was consistent across Poland (*B* = 0.47, *p* < 0.05) and Romania (*B* = 0.60, *p* < 0.01), with the highest value in Slovenia (*B* = 0.96, *p* < 0.001). Traditional media information was the second by size (*B* = 0.42, *p* < 0.001), but present as statistically significant only in Poland (*B* = 0.52, *p* < 0.01) and Romania (*B* = 0.51, *p* < 0.01). Close to this by size was the effect of trust in official sources (*B* = 0.34, *p* < 0.01). It was significant for Romania (*B* = 0.67, *p* < 0.001) and Slovenia (*B* = 0.52, *p* < 0.05), but not for Poland. Digital media was also a significant predictor; however, only in the general sample (*B* = -0.19, *p* < 0.05) and in Slovenia (*B* = -0.41, *p* < 0.05).

The results of the regression in M2 indicated a substantial additional increase in the model fit (Cox-Snell *R*^*2*^ = 0.47, Nagelkerke *R*^*2*^ = 0.63, Model χ^2^ = 559,474, df = 23, *p* < 0.001). Almost all the information source effects disappeared after attitudes and mentality were included in M2, except for trust in the traditional media information in the general sample (*B* = 0.26, *p* < 0.001). Mistrust of the vaccine benefit was significant in the general sample (*B* = -0.89, *p* < 0.001), and in all countries: Poland (*B* = -0.93, *p* < 0.001), Romania (*B* = -1.09, *p* < 0.001), and Slovenia (*B* = -1.01, *p* < 0.001). Concerns about commercial profiteering were only present in the general sample (*B* = -0.34, *p* < 0.01), and in Poland (*B* = -0.69, *p* < 0.01). Mixed preference for the vaccine type was a stronger predictor (*B* = 1.68, *p* < 0.001) than only mRNA vaccine type preference, strongest in Poland (*B* = 4.61, *p* < 0.05) and Romania (*B* = 2.27, *p* < 0.001), and absent in Slovenia.

The results for all hypotheses are summarized in Table 4.

**Table 4:** Summary of results

HYPOTHESIS	general sample	Poland	Romania	Slovenia
**H1a: Negative attitudes towards vaccination predict negatively the intention to take the COVID-19 vaccine.**	(-) Mistrust of vaccine benefits (-) Concerns about commercial profiteering	(-) Mistrust of vaccine benefits (-) Concerns about commercial profiteering	(-) Mistrust of vaccine benefits	(-) Mistrust of vaccine benefits
H1b: Conspiracy mentality negatively predicts the intention to take the COVID-19 vaccine.	-	-	-	-
**H2: The preference for mRNA vaccines positively predicts the intention to take the COVID-19 vaccine.**	(+)	(+)	-	-
H3a: There is a relationship between trust in COVID-19 information from relatives and friends and the intention to take the COVID-19 vaccine.	-	-	-	-
**H3b: Trust in COVID-19 information from medical professionals and scientists positively predicts the intention to take the COVID-19 vaccine.**	(+)	(+)	(+)	(+)
H3c: Trust in COVID-19 information from religious leaders positively predicts the intention to take the COVID-19 vaccine.	-	-	-	-
**H3d: Trust in COVID-19 information from official sources positively predicts the intention to take the COVID-19 vaccine.**	(+)	-	(+)	(+)
**H3e. Trust in COVID-19 information from traditional media positively predicts intention to take the COVID-19 vaccine.**	(+)	(+)	(+)	-
**H3f. Trust in COVID-19 information from digital media negatively intention to take the COVID-19 vaccine.**	(-)	-	-	(-)

Note. The supported hypotheses are bolded

## Discussion

According to our research conducted in April 2021, 62% of Poles, 33% of Romanians, and 40% of Slovenians who were unvaccinated had the intention to get vaccinated. Combining this with vaccination data in April 2021 [16], this indicates that 5 months later we could expect Poland to reach a vaccinated population (with at least one dose of COVID-19 vaccine) level of 40%, Romania at 28%, and Slovenia at 27%. For Poland, this is almost 12 points below the actual vaccination rate (52%), and for Slovenia, it is 24% points below the actual vaccination rate (51%), while for Romania, it almost exactly matches the vaccination rate five months later (28%).

These results could be explained by the changes in vaccination attitudes after April 2021, which might indicate that Poland and especially Slovenia have had a vaccination campaign that, to some extent, affected public opinion. However, there was a constant struggle to combat misinformation while focusing on preventing the transmission of the virus. Even in April 2021, based on the results of our vaccination intention survey, it was anticipated that the herd-immunity goal would not be reached by the upcoming autumn COVID-19 wave without a more target-focused and effective vaccination strategy, especially in Romania. Hence, our study aimed to offer insights into the factors influencing COVID-19 vaccination intention in the Central and Eastern European context. Subsequently, we aimed to derive practical implications to inform region-specific health campaigns in Central and Eastern European countries.

In line with our assumptions, some of the negative attitudes towards vaccination negatively predicted the intention to receive the COVID-19 vaccine (H1a supported). Specifically, mistrust of the vaccine’s benefits was a significant predictor in all three countries, while concerns about commercial profiteering only had a significant effect in Poland. No effect could be demonstrated for concerns about possible unforeseen effects or the preference for natural immunity. The study shows that at the onset of the pandemic, the fear that vaccines do not bring benefits, and, on the contrary, they can be potentially harmful seems to be one of the most influential factors for the vaccination decision. The fact that Poland was the only country where concerns about commercial profiteering were a significant predictor may be the result of the general distrust of pharmaceutical companies and their activities. According to Ipsos, only 28% of Poles think that pharmaceutical companies are trustworthy [41]. Regarding hesitance towards COVID-19 vaccination, their main concern was a lack of trust in pharmaceutical companies related to the too rapid introduction of a product to the market [42].

We did not confirm the relationship between a conspiracy mentality and the intention to take the COVID-19 vaccine (H1b not supported). The absence of a relationship may stem from our assessment of this phenomenon as general beliefs not specifically associated with theories related to COVID-19 and vaccination. Some research also suggests that, rather than the direction proposed by us, the relationship may be the opposite. Initial hesitancy about being vaccinated may motivate people to seek reasons not to get vaccinated, which they may find in COVID-19 conspiracy theories [43, 44].

We confirmed that a preference for mRNA vaccines positively predicts the intention to vaccinate in a general group (H2 supported). However, we also discovered that countries differed in terms of preferences for the type of vaccine. Only in Poland, the exclusive preference for mRNA vaccines positively predicted the intention to vaccinate. This aligns with previous Polish studies that revealed Poles widely accepted mRNA vaccines [17]. In Poland, mRNA vaccines received more attention from the media and expert groups, shedding light on their mechanisms of action. This likely contributed to a higher level of acceptance of this type of vaccination. On the contrary, AstraZeneca (vector vaccine) received a less favourable reception among the Polish population due to emerging information about possible side effects occurring after the first dose (with mRNA vaccines, these effects were also present but tended to occur more frequently after the second dose) [17]. In Romania, only the mixed vaccine preference had a significant effect, while in Slovenia, neither effect (for mRNA preference nor mixed preference) was significant. The lack of effect in Slovenia might be due to the fact that at the start, mRNA vaccines were exclusively administered to individuals aged 65 and over, with the AstraZeneca vaccine being limited to those aged between 18 and 64 [45]. Official recommendations for mRNA vaccines in Slovenia only began in October 2021 [46].

Regarding trust in information sources, consistent with findings from some prior studies [22], we did not confirm the relationship between family and friends’ opinions and vaccination intention (H3a not supported). Additionally, religious sources did not seem to exert a significant impact on vaccine intention in this setting (H3c not supported). These results suggest that at the onset of the pandemic, other information sources may be more influential in shaping vaccination intentions. In line with that, we discovered that trust in medical professionals and scientific sources in all three countries positively predicted vaccination intention (H3b supported). However, the lowest coefficient was observed in Poland. In 2021, a YouGov international survey revealed that Poland is the only country where healthcare professionals are not the most trusted group for COVID-19 information [47]. Research on the impact of trust in medicine on Polish citizens’ adherence to recommended behaviors showed that 63.8% of Poles express low or moderate trust in healthcare professionals, vaccines, and medicines [48]. Conversely, the highest coefficient was observed in Slovenia, where trust in doctors is high, as confirmed by studies conducted during the pandemic, in which respondents expressed the most trust in doctors and pharmacists (76%) [49].

What is more, trust in information from official sources also positively predicted intention to vaccinate (H3d supported). This is consistent with previous studies showing that trust in governmental authorities positively predicted various positive health behaviors [50, 51]. However, in Poland, this effect was not significant. Polish society appears to distrust official sources, a trend supported by YouGov studies where Poland showed one of the lowest levels of trust in COVID-19 information from official sources [47]. In our study, the highest coefficient was observed for Romania, which is consistent with other studies indicating that government websites were the most trusted source of information for Romanians during the period studied [52].

Regarding media, our research indicates that individuals who trust information from traditional media are more likely to receive the vaccine (H3e supported). These findings align with previous studies [34], emphasizing the crucial role of traditional media, including television, radio, and the press, in disseminating essential information about the pandemic. Traditional media is generally perceived as more reliable due to its higher level of control and oversight, often in collaboration with official authorities [53]. However, the effect of traditional media did not manifest in Slovenia, which can be attributed to a general distrust of mass media among Slovenians. As studies suggest, this scepticism during the pandemic may stem from contradictions among different media outlets in Slovenia, partly attributed to their diverse political backgrounds [54].

We also demonstrated that trust in digital media negatively predicts the intention to vaccinate (H3f supported), highlighting the capacity of the internet and social media to disseminate misinformation and amplify vaccine hesitancy [34]. However, this was true only for Slovenia. Studies suggest that an important factor in protecting from misinformation on social media is media literacy, and other research shows that Slovenia belongs to of well-performing countries in terms of media literacy [55].

However, after including attitudes and beliefs in the model, the effect of all information source variables on the intention to vaccinate was not significant. It appears that a general mistrust of vaccines is the key factor influencing vaccination intention. This is supported by other research indicating that trust in vaccine effectiveness is strongly associated with the intention to be vaccinated against COVID-19 [56].

### Limitations and future research

There are important limitations to this research. The study concerned three selected countries in Central and Eastern Europe. Future research should be extended to other countries in the region. The study was conducted on a group of previously unvaccinated people. This made it impossible to investigate the motives of those who had already been partially or fully vaccinated during the period considered. Future research may include both vaccinated and unvaccinated respondents for a better understanding of the hesitancy towards vaccination.

Another limitation is the divergences in the methodology due to the country-specific sampling approaches, specifically for Romania, where convenience sampling was used, which can induce a large selection bias. Moreover, we should consider language specifics and differences in the online software tools used for data collection. In addition, the sample sizes in Poland and Romania were much lower than those in Slovenia, which means a larger margin of error in the estimates. The results of multivariate analysis are based on a cross-sectional design and thus should be taken as exploratory and correlational.

Based on previous research, we included numerous control variables to ascertain the net effect of the key variables under investigation; however, we cannot exclude the possibility of any remaining common factor. Additionally, without longitudinal or experimental data, the causal order of the variables cannot be ascertained.

### Practical implications

Based on the results and conclusions of our study, we provide some practical implications to assist in implementing more effective and efficient health messaging strategies and campaigns targeting groups reluctant to receive the COVID-19 vaccine in Poland, Romania, and Slovenia. Since the attitude based on mistrust of vaccines has proven to be a strong predictor of the intention to get vaccinated, communication campaigns about vaccinations should focus on presenting the benefits of vaccination. Various recent studies also support this notion [56]. Instead of combating conspiracy beliefs to increase vaccine acceptance, our results suggest focusing on disseminating positive information about the effectiveness of vaccines and their benefits. However, as for Poles concerns about commercial profiteering predict vaccination intention, it is also important to present messages aiming to reduce the distrust of pharmaceutical companies and their activities. This involves showing transparency in the operations of these companies and promoting and informing about therapeutic successes. This is also consistent with another finding from our study regarding the preference for a type of vaccines, indicating that effective communication about producers and vaccine types is also crucial in shaping public attitudes, especially at the beginning of the pandemic. When new drugs preventing the spread of a pandemic emerge, a lack of proper information and appropriate promotion can deepen vaccine hesitancy [57].

In terms of social influences our study demonstrates the importance of medical professionals and scientists information. It is crucial to promote scientific evidence and present educational campaigns by professionals, as they are considered the main source of influence on the key determinants of vaccination. Also, official sources are influential for obtaining reliable information [31, 32]. However, in Polish society, there is no relationship between trust in official sources and the intention to vaccinate. This lack of trust in official sources might suggest a danger to health behaviors. Therefore, it is important to work on building trust in the government and identifying communicators within the government who are socially trusted, especially in Poland.

Additionally, public health communicators must decide which media platforms to use for sharing information on COVID-19, considering their credibility. Traditional media appears to be a better choice than social media, given the spread of misinformation and the influence of filter bubbles, which can affect people’s opinions [34]. Nonetheless, in the context of Slovenia, with no significant effect on traditional media and a significant negative effect on digital media information, the communication dynamics present an intriguing case with distinctive patterns. The results suggest that relying on official sources for communication, extending beyond the scope of digital media alone, emerges as the optimal current strategy for vaccination in Slovenia.

To sum up, considering the array of solutions implemented worldwide in response to the pandemic threat, it is imperative to direct attention primarily towards public health services. The crucial aspect of health policy guidance involves the necessity to tailor the solutions implemented, and more importantly, the narrative and communication with society, to the prevailing social, cultural, and historical conditions in each country. In order to achieve this, it is essential to delve into the factors influencing COVID-19 vaccination intention, which, as indicated by our research, could provide insights for communication strategies.

## Data Availability

The data used in this research will be made publicly available in the Slovenian Social Science Data Archive (ADP) [38].

## Footnotes

1 As of 16.04.2021, the share of COVID-19 vaccine doses was the highest for the mRNA BNT162b2 vaccine (Poland: 71%; Romania: 81%; Slovenia: 67%), followed by the vector vaccine AZD1222 (P: 21%; R: 11%; S:22%), Moderna mRNA-1273 (P: 7%; R: 8%; S: 10%) and the Ad26.COV2.S vector vaccine (P: 0.3%; R: 0%; S: 0%). As of 17.09.2021, the share of COVID-19 vaccine doses was still the highest for BNT162b2 (P: 71%; R:77%, S: 67%). The AZD1222 vaccine share was lower than in April (P: 15%; R: 9%; S: 17%), while Moderna’s mRNA-1273 share remained approximately the same (P: 9%; R: 7%; S: 10%) and the share of Ad26.COV2.S was higher than in April (P: 5%; R: 7%; S: 6%) [16].
